# Diabetic Pneumopathy–A New Diabetes-Associated Complication: Mechanisms, Consequences and Treatment Considerations

**DOI:** 10.3389/fendo.2021.765201

**Published:** 2021-11-25

**Authors:** Stefan Kopf, Varun Kumar, Zoltan Kender, Zhe Han, Thomas Fleming, Stephan Herzig, Peter P. Nawroth

**Affiliations:** ^1^ Department of Medicine I and Clinical Chemistry, University Hospital Heidelberg, Heidelberg, Germany; ^2^ German Center for Diabetes Research (DZD), Munich-Neuherberg, Germany; ^3^ European Molecular Biology Laboratory, Advanced Light Microscopy Facility, Heidelberg, Germany; ^4^ Institute for Diabetes and Cancer, Helmholtz Center Munich, Munich-Neuherberg, Germany; ^5^ Joint Heidelberg-Institute for Diabetes and Cancer (IDC) Translational Diabetes Programme, Helmholtz-Zentrum, Munich, Germany

**Keywords:** diabetes, pulmonary fibrosis, senescence, persistent DNA damage signaling, hyperglycemia

## Abstract

Patients with diabetes are over-represented among the total cases reported with “idiopathic” pulmonary fibrosis (IPF). This raises the question, whether this is an association only or whether diabetes itself can cause pulmonary fibrosis. Recent studies in mouse models of type 1 and type 2 diabetes demonstrated that diabetes causes pulmonary fibrosis. Both types of diabetes trigger a cascade, starting with increased DNA damage, an impaired DNA repair, and leading to persistent DNA damage signaling. This response, in turn, induces senescence, a senescence-associated-secretory phenotype (SASP), marked by the release of pro-inflammatory cytokines and growth factors, finally resulting in fibrosis. Restoring DNA repair drives fibrosis into remission, thus proving causality. These data can be translated clinically to patients with type 2 diabetes, characterized by long-term diabetes and albuminuria. Hence there are several arguments, to substitute the term “idiopathic” pulmonary fibrosis (IPF) in patients with diabetes (and exclusion of other causes of lung diseases) by the term “diabetes-induced pulmonary fibrosis” (DiPF). However, future studies are required to establish this term and to study whether patients with diabetes respond to the established therapies similar to non-diabetic patients.

## Introduction

### Current Stage of the “Pneumopathy” as a Potential Diabetic Complication

In 1976, Schuyler et al. published the first paper suggesting that the lung might be a target organ for diabetic complications (1). Since then, many publications have reported the association of type 1 or type 2 diabetes in several pulmonary diseases (2–9). Not surprisingly, there were many conflicting results, potentially explained by the heterogeneous nature of diabetes and its metabolic comorbidities, but also by the different techniques used for characterizing the lung physiology and the basal characteristics of the patient cohort analyzed. Nevertheless, the field has significantly developed overtime. Until now, there is almost no pulmonary disease that has not been brought into the context of diabetes, most recently a more severe course of a SARS-CoV-2 infection (**Box 1**) (34). More importantly, despite reduced alveolar gas exchange in patients with type 2 diabetes (35, 36), a clinically proven accelerated decline in lung function in diabetic patients (37–42), the pathologically proven abnormalities of alveolar capillaries and the role of pulmonary autonomic dysfunction (35, 43, 44), the issue of diabetes-induced pulmonary dysfunction has not gained the attention it deserves. Since most studies describe an association only, the lack of proven causality thus far precluded the acceptance of the lung as a bonafide target of diabetic complications.

Box 1 **Pulmonary diseases potentially associated with diabetes [reviewed in REF. (3)].**

- Idiopathic pulmonary fibrosis (1, 10–13)
- COPD (14–17)
- Asthma (18–22)
- Pulmonary hypertension (23–26)
- Lung cancer (27–29)
- More severe course of SARS-CoV2 (30)
- Increased risk of inflammatory lung disease (such as tuberculosis, pneumonia, autoimmune and connective tissue diseases) (31–34)

Similarly, the association with insulin resistance (making a causal relationship even more likely) could not change the oversight of this research area in diabetes, nor did the finding of reduced pulmonary function as a function of increased HbA1c convince the mainstream diabetologists (38, 45). This also holds for the well-known changes in cellular properties, potentially contributing to lung disease in patients with diabetes. Among these, oxidative stress (37, 43), formation of the inflammasome (46), WISP mediated IL-6 dependent proliferation of human lung fibroblasts (47), activation of metalloproteinases (48) and others have been suggested to contribute to lung disease. In this context, accumulation or co-localization of advanced glycation end-products with cell surface RAGE has been described (49). Altogether these might play a role in the accelerated aging of human collagen in diabetes (50). Inflammation, the AGE-RAGE interaction, diabetes-induced platelet-endothelial cell interaction, changes in NOS activation, reduced NO bioavailability, activation of the JAK/STAT pathway, and release of growth factors have been described to occur in diabetes (36). But many of these studies lack a clear demonstration of the cause-effect relationship. Thus, despite a high degree of plausibility, the definite proof of diabetes-related activation of a mechanism causing a specific lung disease is still limited. Therefore, it is not surprising that a recent comment pointed to look deeply into diabetic pneumopathy as a diabetic complication (9).

Reviewing the field of diabetes and lung disease, it becomes evident that there is a large number of exciting data and many clinical observations supporting an important role of the lung as a diabetic target. This is contrasted by the general neglectance of “diabetic pneumopathy” as an important topic in the field: It is hardly presented at diabetes meetings, very rarely in educational sessions on diabetic complications (30, 51). Thus, it is not a generally accepted exciting topic in diabetes research and clinical practice. This might, in part, be due to a large number of observational studies compared to the lower number of studies proving a cause-effect relationship.

Most importantly, from our point of view, the lack of combinatorial studies which involves *in vitro*, animal-based *in vivo* models and clinical cohorts, not just limits the understanding but also blocks the deep insights this area, which diabetologists require. However, in one study (5, 52) there is now evidence that hyperglycemia is causatively linked to the onset and progression of pulmonary fibrosis in patients with diabetes, suggesting that these patients should be classified as patients with diabetes-induced pulmonary fibrosis (DiPF).

## Requirements for Recognition as a Diabetic Complication

As stated above, the simple association of an increased risk of lung disease in patients with diabetes does not necessarily mean that the lung disease is caused by diabetes. It is well possible that prenatal or early in life injuries prime an individual to develop diabetes and a pulmonary disorder. Similarly, there are various socio-economic factors that directly play an important role in the progression of diabetic complications, which also need to be considered. Thus, neither the sequelae of events nor its correlation can prove causality. Ideally, a study in which patients are divided into two groups, one with good and one with poor glucose control, would give insight into a causal relationship. However, if an agent such as insulin, known to potentially affect lung function (3, 53–55), is used for treatment, the endogenous effects of insulin might outweigh the impact of better glucose control. Given this difficulty and the lack of an intervention study with the prime goal of studying the effect of glucose control by insulin or oral glucose-lowering drugs, other criteria are needed to establish a certain disease as a complication caused by diabetes.

The criteria used in this review include: (i) The clinical association shown in more than one study, (ii) the similarity in type 1 and type 2 diabetes, (iii) the proof of a cause-effect relationship in animal models of type 1 and type 2 diabetes, which includes its reversibility by interference with an assumed pathogenic mechanism and if possible, and (iv) the translation of the *in vitro* data and experimental diabetes findings into the human situation. The following part of this review will determine whether this holds in the case of pulmonary fibrosis in patients with type 1 and type 2 diabetes. The reader might decide, whether there are sufficient data to state that there is diabetes-induced pulmonary fibrosis (DiPF).

## Clinical Aspects, Diabetes Treatment and Their Cross Implications on Lung

Several of the drugs used in the treatment of diabetic complications have been described to affect lung function. For example, metformin has been associated with increased survival rates in patients with lung cancer and pulmonary inflammation, including radiation pneumonitis (56, 57), and reduced exacerbations of asthma (58, 59). In contrast, some studies point to an association of insulin use with lung cancer (53, 54), an increased risk of asthma (60), and alveolar microangiopathy (61). One study showed a protective role of glibenclamide in asthma development (62), GLP-1 agonists a decreased COPD exacerbation and reduced mortality (63), and SGLT-2 inhibitors may induce pulmonary artery smooth muscle cell relaxation (64).Therefore, substantial open questions remain concerning the beneficial or detrimental effects of the drugs used to treat diabetes on lung function.

## Diagnosis and Idiopathic Pulmonary Fibrosis

Idiopathic pulmonary fibrosis (IPF) is a term used for patients in whom pulmonary fibrosis is diagnosed. However, none of the well-known etiologies explaining the development of fibrosis can be found (61). The problem starts with the difficulties in diagnosis, since different diagnostic criteria have been suggested by other investigators and organizations (65, 66). The main point of discussion was whether subtle histopathological differences between usual or nonspecific interstitial pneumonia should be used for the differentiation of distinct disease entities, since different diseases with defined etiologies may result in the same histological picture. Therefore, others state that IPF is a chronic, progressive and irreversible lung disease of unknown origin (51, 67). IPF is now accepted to be caused by genetic and environmental risk factors with repetitive local micro-injuries to the alveolar epithelium (61), leading to aberrant epithelial-fibroblast communications and a myofibroblast-dependent thickening of matrix and remodeling of lung interstitium (68, 69).

Based on radiological and histological criteria, IPF is diagnosed by the identification of a pattern of interstitial pneumonia in patients without evidence of an alternative cause (51, 66, 68–70). Thus, the exclusion of other diseases is a challenge for the clinician. Furthermore, several other illnesses linked to IPF have similar appearances in x-ray and CT scans. This includes asbestosis, chronic hypersensitivity pneumonitis, connective tissue diseases, drug toxicity and others. A careful patient history covering its exposition to environmental challenges, medications, socio-economic factors, and symptoms for cardiac disease could play an important role in diagnosis. In addition to clinical symptoms, such as breathlessness, a reduced six-minute-walking test, lung function tests including bodyplethysmography, diffusion capacity measurements combined with a multi-detector CT scan and maybe spiroergometry or bronchoalveolar lavage and lung biopsy, might help to make the correct diagnosis (51, 66, 70–74). The clinical course of patients with IPF is heterogeneous, with a median survival of 2.5-3.5 years post-diagnosis (68, 69). Many different mechanisms potentially leading to IPF have been described, and it includes steady inflammation, genetic and environmental interactions leading to epithelial injury, changes in the unfolded protein response, absence of type 1 pneumocytes, differentiation of fibroblasts to myofibroblasts, activation of matrix metalloproteinases, changes in angiogenesis, and maladaptive repair (65, 68, 69, 75). Since all of these mechanisms are observed during the course of type 1 and type 2 diabetes, it is plausible to study the association of pulmonary fibrosis with type 1 and type 2 diabetes.

## Pulmonary Fibrosis in Patients With Diabetes

Several studies have reported an association of type 1 and type 2 diabetes with restrictive lung disease and pulmonary fibrosis (2–5, 7, 10, 11, 23, 76–79). While several authors found a decline in lung function in patients with diabetes (38–42), this does not yet prove that this decline is due to pulmonary fibrosis. In 1990, Lange and coworkers found that during a 5-year observation period, that the forced expiratory volume in one second and forced vital capacity declined faster in patients with type 2 diabetes (39), which was supported by the ARIC study (79). Enomoto et al. calculated that the adjusted odds ratio for IPF due to smoking was 5.4, while it was almost the same, namely 4.06 for diabetes (61), suggesting that diabetes may be an independent risk factor for IPF. This fits well with several studies showing that the prevalence of diabetes among patients with IPF is higher than in the general population or in other lung diseases (7, 10, 18, 76, 80–82). The meta-analysis by Vanden Borst et al. (8) provided clear evidence of an association between diabetes and restrictive lung disease, probably fibrosis. The relation of pulmonary fibrosis to type 1 diabetes became likely because restrictive lung disease was correlated with diabetic nephropathy (61), known to be associated with renal fibrosis.

**Figure 1:**
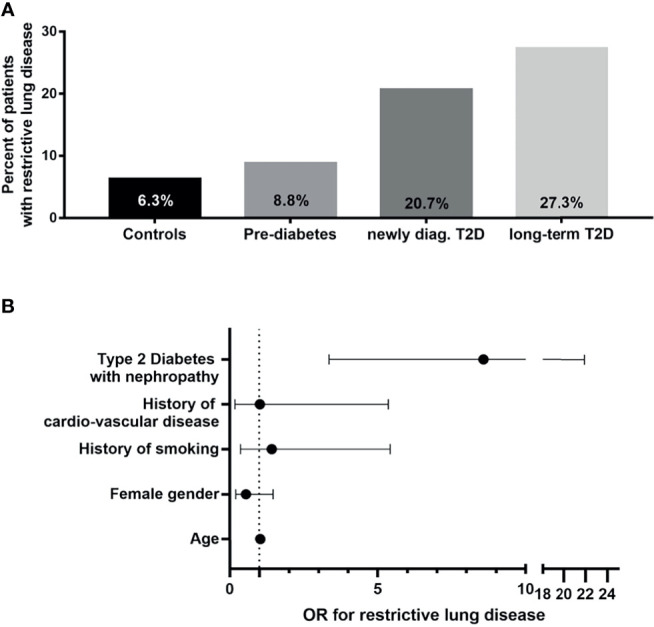
Clinical characterization of diabetic patients with restrictive lung disease. **(A)** Percentages of patients with restrictive lung disease shown in bar graphs according to group [as shown (5)]. **(B)** Calculated odds ratios (OR) with 95% confidence interval for restrictive lung disease plotted on x-axis for selected clinical parameters in patients with type 2 diabetes and albuminuria as compared to non-diabetic patients [modified as described earlier (5)].T2D, type 2 diabetes; OR, odds ratio.

Furthermore, a relation to glycemic control was observed in type 1 diabetes. A study from Korea showed that metabolic syndrome increased the odds ratio for restrictive lung disease (diagnosed by FVC<80%, FEV1/FVC<0,7) to 1.4 (83), indicating that not only type 1 diabetes but even early stages of type 2 diabetes, affect the lung. An association of HbA1c with a restrictive spiroergometric pattern (61) supported the view that restrictive lung disease is a diabetic complication. Besides, patients with diabetes and restrictive lung disease have a worse prognosis (67, 84). A recent study shed additional light on this topic by using a combined approach of clinical symptoms, clinical tests, including a six-minute-walking-test, comprehensive lung function tests, and high-resolution CT, as well as histology in patients with type 2 diabetes (5). In this cross-sectional study, 48 non-diabetic patients, 68 patients with prediabetes, 29 patients with newly diagnosed type 2 diabetes, and 110 patients with long-term diabetes were thoroughly examined for metabolism, all known diabetic complications, but also for clinical parameters, such as breathlessness and 6-minute walking test. Some patients underwent a multidetector computed tomography, and in some patients, histology was also performed. The study showed that breathlessness in combination with restrictive lung disease was found in 9% of the patients with prediabetes, 20% of patients with newly diagnosed diabetes, and 27% of patients with long-term type 2 diabetes (**Figure 1A**).

Most importantly, the presence of albuminuria increased the risk for restrictive lung disease (OR 8.57) as compared to non-diabetic patients (**Figure 1B**). Multidetector computed tomography confirmed interstitial lung disease, and histological analysis confirmed the presence of increased alveolar septal fibrosis. There was a relation between clinical symptoms and the severity of lesions observed in CT scans. However, there was no typical or uniform morphological appearance in this small series of CT scans since two patients had subtle and mild intra- or interlobular reticulations, other sub-pleural intralobular reticulations, and ground-glass opacities. In contrast, the patient with the most severe clinical symptoms showed marked sub-pleural intralobular reticulation. Recently, one study with a follow-up of three years was published (61). Here, the decline of lung function in 3 years was not significantly higher in patients with type 2 diabetes than in non-diabetic patients. Furthermore, having more patients studied, the progression of clinical symptoms (breathlessness and 6-minute walking test) correlated well to the CT scan. Of note, even in this more extensive series, no diabetes-specific CT scan pattern could be detected. Thus, more comprehensive studies involving diabetic patients with IPF, but excluding those with other pulmonary and/or cardiac disease, are needed to determine whether there exist any diabetes-specific signatures in high-resolution CT scans of diabetic patients with IPF.

Overall, both studies together strongly suggest to systematically study diabetic patients for pulmonary fibrosis. This includes a detailed history, the 6-minute-walking-test, and, if indicated, a multi-detector high-resolution CT scan (**Box 2**). These data make it very likely that diabetes is indeed responsible for developing pulmonary fibrosis, especially since markers of glycemic control were predictive of a declining lung function in this study. Whilst the data are convincing for patients with type 2 diabetes, studies using clinical, functional, and CT scans are missing for type 1 diabetes. For the final proof, a randomized controlled study of poor and intensified glycemic control patients with type 1 diabetes with insulin and patients with type 2 diabetes using oral anti-hyperglycemic drugs is required. It would help to learn more about the assumed pulmonary side effects of insulin. However, at the moment, the likelihood of this kind of study is relatively low. As long as such a clinical study is lacking, animal studies will have to provide the final proof of the existence of DiPF.

Box 2 **Work up and indication for work up.**

Patients with:

- Long-standing diabetes and/or albuminuria
- Breathlessness and exclusion of cardiac disease (NT-pro-BNP, cardiac ultrasound, exclusion of coronary heart disease) or other known lung diseases (e.g. pneumoconiosis, extrinsic allergic alveolitis, Radiation-induced lung injury)
- 6-minute-walking-test with a reached distance below 400 meters (normal persons would reach between 700-800 meters) (85)
- Increased dyspnea during the walking test (quantified by using the modified BORG-scale ranging from 0 to 10) (86)
- Suspect lung function testing with reduced forced vital capacity (FVC) <80%, reduced total lung capacity in bodyplethysmography (TLC-B) <80%, and reduced single breath diffusion capacity (SB-DL_CO_) <80% in the presence of normal or increased Tiffineau-index (FEV1/VC) (71, 87, 88).

## Socio-Economic Consideration in Diabetes-Associated IPF Diagnosis

Like other infectious diseases, the onset and progression of diabetic complications are directly influenced by the socio-economic status of the patients. Among the common socio-economic factors, such as education level, income and occupation are directly linked to diabetic complications (89–91). Persistent ignorance or poor understanding of future complications is also linked to early school dropouts and the premature onset of late-stage complications (89, 90). However, such detailed studies have yet not been described in the context of diabetic lung fibrosis. Still, in several studies, it has been shown that low socio-economic groups are mostly associated with insufficient health care facilities and shows an inverse relationship to lung fibrosis, asthma, pulmonary hypertension and other chronic respiratory diseases to the socio-economic status. Thus current scenario of diabetic complications needs a comprehensive investigation by pairing the socio-economic factors to the onset of diabetic pneumopathy.

## Pathophysiological Studies and Experimental Diabetes Models

Several studies in mice and rats have shown that in models of type 1 or type 2 diabetes, pulmonary fibrosis is prominently evident. The first study, published in 1979, showed streptozotocin (STZ)-mediated changes in lysyl-oxidases (92). Later studies in diabetic rats demonstrated the appearance of pulmonary fibrosis and, most importantly, for the support of diabetes-induced pulmonary fibrosis, they demonstrated in STZ treated mice an almost complete normalization of lung fibrosis by insulin treatment (93). This directly indicates that hyperglycemia causes pulmonary fibrosis, which extends our view on DiPF from an association to a causative effect of diabetes. This is seen in STZ treated mice. But since STZ is an agent known to have pro-inflammatory and DNA damaging effects, it is important that similar effects are also observed in different models, such as the OVE26 transgenic mouse, which develops diabetes without the addition of a toxin and can be kept without insulin administration (94). The fact that pulmonary fibrosis was not only seen in mice and rats but also in diabetic cats (95) makes it likely that these findings are not attributed to artefacts of animal models but rather confirmed and bona fide complications of diabetes (96, 97).

**Figure 2:**
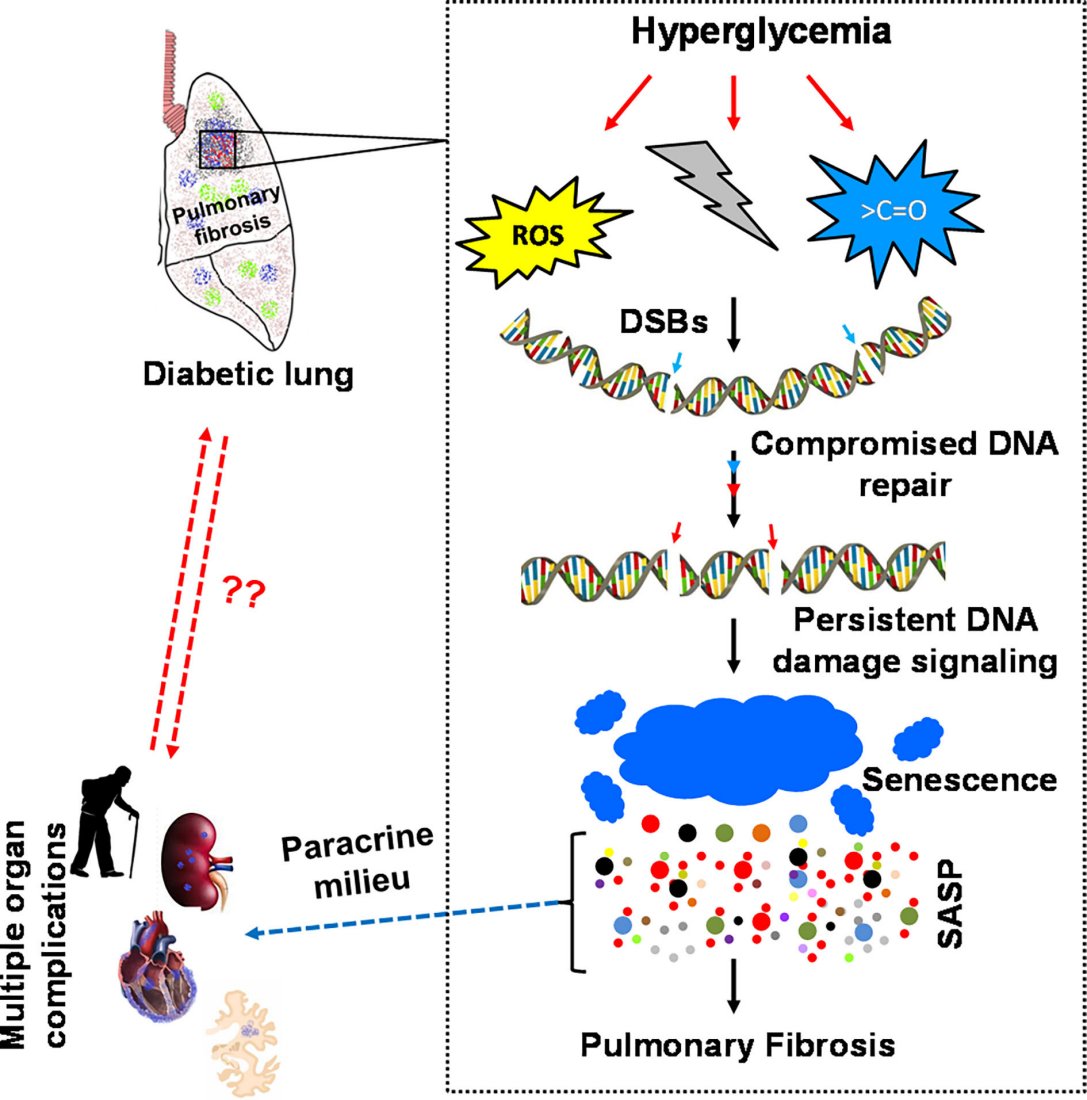
Diabetes-associated persistent DSB signaling, senescence and SASPs modulates the normal repair of idiopathic pulmonary fibrosis. Diabetes-associated perturbed cellular metabolism is cumulatively linked to diabetic pneumopathy. Elevation of ROS, disturbed NAD^+^/NADH equilibrium, and non-specific modifications of active biomolecules affect the integrity of the genome and the kinetics of DNA repair. The absence of timely DNA repair activates persistent DNA damage signaling, followed by an irreversible cell cycle arrest, cellular senescence and senescence-associated secretory phenotype (SASP). The SASP releases various pro-inflammatory cytokines such as IL-1α/β, IL-6, IL-8, TGF-β, ICAM, Mcp1 & TNF-α. These cytokines act autocrine and paracrine by altering the cellular homeostasis to maladaptive genetic transformations resulting in severe inflammation and organ fibrosis. The cytokines released from the SASP zone, such as IL-6, IL-8 and TGF-β, activate the fibrotic program *via* the JAK-STAT pathway. Therefore, the accumulated ECM compromises the functional integrity of diabetic lungs and transforms the parenchymal organ to idiopathic pulmonary fibrosis.

Thus, reversibility by glucose control and appearance of pulmonary fibrosis in various animal models and various species makes a strong point for the existence of DiPF. However, one fact is still missing, namely, the detailed description of a pathogenic mechanism, which stops or even reverses pulmonary fibrosis in an animal model and at the same time also exists in patients with diabetes. Aging is also a risk factor for IPF, and it is not surprising that cellular senescence plays a role in fibrotic pulmonary disease (98). Furthermore, several signaling pathways such as PI3-Akt, VEGF signaling, TGF-ß, and Wnt/ß-catenin signaling pathways have been shown to be activated in IPF (61). Senescence is intrinsically linked to the so called senescence-associated secretory phenotype (SASP), which involves secretion of growth factors and cytokines, chemokines, and matrix remodelling proteases (98–113). Thus, these senescent cells severely affect their closest environment and systemically the whole organ by its anti-proliferative and pro-inflammatory fibrosis promoting paracrine *milieu *(104, 111, 114–116). In several animal models, senescence has been shown to be directly responsible for the development of fibrosis (**Figure 2**).

## A Conjunction of DNA Damage Response and Senescence in DiPF

Senescence-promoting mechanisms trigger fibrosis, while senescence-inhibiting strategies, including the use of “senolytic drugs”, inhibit fibrosis. This leads to the question of the signals triggering senescence and SASP. DNA double-strand breaks (DSBs) are the most cytotoxic forms of DNA lesions, eliciting a complex series of events classified as DNA-damage- response (DDR), and leading either to error-free homologous recombination repair (HRR) or error-prone non-homologous end-joining repair (NHEJ) (117). HRR requires extensive nucleolytic processing of broken DNA ends, generating single strand DNA-tails (118, 119). In this region, the so-called DNA-damage sensor complex forms, consisting of a protein called Receptor for Advanced Glycation End-products (RAGE) (111, 120–124), an enzyme MRE11, and its cofactors Rad50 and Nbs1 (119). This complex then helps to convert the exonuclease activity of MRE11 to the required endonuclease activity (111). In the absence of timely generation of an endonucleolytic activity of MRE11, SASP inflammation and fibrosis occurs (104, 115, 116, 125). The important role of RAGE as a cell surface receptor promoting diabetic complications has been described previously (125–128). When RAGE is expressed at the cell surface, it can bind as a pattern recognition receptor not only AGE´s, but also other pro-inflammatory mediators, such as S100 proteins and HMGB1 (129). Binding RAGE triggers a cascade of pro-inflammatory reactions, contributing to late diabetic complications. However, the lung is strikingly different from other organs, such as skin. Therefore, it more directly encounters the environmental challenges, thus constitutively expresses large amounts of nuclear RAGE (130–132). Loss of RAGE impairs pulmonary repair mechanisms, which altogether promotes pulmonary fibrosis (111, 129, 133, 134). This is not surprising since other pattern recognition receptors are also involved in DNA repair (133, 135, 136).

## Nuclear RAGE and DNA Repair in Persistent DNA Damage Signaling

Nuclear RAGE is part of the DNA-damage sensor complex and recruits to the site of DNA damage *via* an ATM signaling cascade. At the site of damage, it interacts with the DNA end-resection complex MRN (MRE11, Nbs1 and Rad50). RAGE modulates the end-processing activity of MRE11 from an unwarranted exo- to an endo-nuclease. This explains why RAGE^-/-^ mice suffer from increased senescence, SASP, pulmonary fibrosis, and reduction of lung function (111). Most importantly for the topic of pulmonary fibrosis, reconstitution of RAGE restores DNA DSBs repair, thus reverses pulmonary fibrosis, in part, by promoting efficient RPA2 and CHK1 phosphorylation, required for efficient DNA repair and prevention of senescence (111). In the absence of nuclear and timely phosphorylated RAGE, DSBs signaling can orchestrate the cascade leading *via* senescence and SASP to fibrosis.

These findings shed new light on the mechanisms leading to pulmonary fibrosis in patients with diabetes (99). The health risks of the Western lifestyle reflect the net result of defective defense and repair, affecting many organs, including the lung (137, 138). In diabetes, genotoxic reactive metabolites such as ROS and dicarbonyls challenges DNA integrity and repair (5, 52, 139–145).The ultimate proof that impaired DNA repair is leading to diabetic complications was missing, but its association with diabetic complications has been shown in several studies (77, 143, 146–151). Since most diabetic complications, including in the kidney, are associated with fibrosis, it was likely that increased DNA damage and impaired DNA repair lead to a persistent DNA damage response, senescence, SASP, and fibrosis. Recently, we have shown that exposure of human alveolar type-II cells (but also other cell types) to reducing carbohydrates, such as glucose, ribose, and fructose, known to be associated with increased ROS formation (123, 140), impair the repair of DSBs repair in the presence of etoposide. The reduction of the DNA repair, especially the non-homologous end-joining (NHEJ) repair pathway, correlated with the reducing capacity of the carbohydrates used. Central to this impairment of the DNA repair capacity was the reducing sugar-triggered change in the NAD^+^/NADH ratio, shifting the equilibrium towards a decreased NAD^+^ and increased NADH cofactor pool required for the activity of SIRT and PARP, two important players in the NHEJ repair (152). In the presence of high glucose, the addition of NAD^+^ disrupted the interaction of PARP1 with its inhibitor Dbc-1, thus making PARP1 available for DNA repair. Therefore, reducing sugars impair the NHEJ repair, leading to a DNA damage response, senescence, SASP, and fibrosis. This was also demonstrated in an experimentaltype1diabetesmodel and supported by db/db mice data. Diabetes was associated with increased activation of the DSBs repair pathway, including an increase in oxidative stress, a shift in the NAD^+^/NADH-ratio, senescence, SASP, pulmonary and renal fibrosis.

An AAV-driven expression system for RAGE, which exclusively results in nuclear expression of RAGE, reduces the DNA damage response-driven cascade causing renal and pulmonary fibrosis in experimental diabetes. Most interestingly, while fibrosis was reduced, there was no effect on albuminuria, indicating that microangiopathy and organfibrosis is not causally connected. Since one of the requirements to accept the term DiPF is the similarity of events found in mice and humans. DNA damage response, the extent of senescence, and SASP were also studied in humans with type 2 diabetes (61). Overall, recent data have established that markers of this cascade are excellent predictors of DiPF, not only in a cross-sectional study but also in a prospective three years study (61). Thus, the cascade leading to the persistent DNA damage response, senescence, SASP, and fibrosis occurs not only in experimental diabetes but similarly in diabetic patients.

### May we use the term DiPF?

The evidence that pulmonary fibrosis is a bona fide diabetic complication is based on the following arguments:

1. Various animal species develop pulmonary fibrosis.
2. Pulmonary fibrosis is seen in experimental type 1 and type 2diabetes.
3. The prevalence of pulmonary fibrosis is higher in humans with type 1 or type 2 diabetes.
4. In humans, the decline of lung function and the presence of pulmonary fibrosis are linked to glycemic control.
5. In experimental diabetes, glucose control alleviates the degree of pulmonary fibrosis.
6. The DNA damage driven cascade leading to senescence, SASP, and fibrosis can be observed in experimental diabetes as well as in humans.
7. At least in experimental diabetes, restoring DNA repair not only stops but rather reverses DiPF.

A prospective, randomized controlled clinical trial to prove the role of glucose control for the development and progression of pulmonary fibrosis in type 1 and type 2 diabetes has yet to be conducted. However, the evidence presented above argues for the existence of DiPF as a bonafide diabetic complication.

## Open Questions

There are many open questions, which require intensive research. This includes a better description of the natural course of DiPF, the characterization of the patients with rapid progression, the differences between type 1 and type 2 diabetes, and of course, the study of therapeutic options: For example, is physical exercise as effective in patients with diabetes as in non-diabetic patients with IPF (153, 154)? Which drugs can be used, and are their effects and side-effects similar in patients with type 1 and type 2 diabetes, as in non-diabetic patients? Based on the patho-mechanisms described above, which strategies can be used to improve and maintain DNA repair or is the repair capacity entirely genetically determined and cannot be subject to treatment (143)?

## Outlook

This review has collected evidence for the existence of diabetes-induced pulmonary fibrosis as a bona fide diabetic complication. Thus, DiPF should obtain the same attention as other complications of the kidney, eye, nerve, vessel, and heart. Therefore, more intense training of clinicians in the history and diagnosis of DiPF warrants its inclusion into educational sessions, textbooks, IPF and national, as well as international diabetes association guidelines.

## Author Contributions

SK, VK and TF researched data for the article. SK, VK, ZK, TF, SH and PPN contributed substantially to discussion of the content. PPN and VK wrote the article. SK VK and ZH created the final figures. All authors reviewed and edited the manuscript before submission.

## Funding

This study was supported by the Deutsche Forschungsgemeinschaft (SFB 1118 & GRK 1874-DIAMICOM), DZD and the Helmholtz Cross Program Topic Metabolic Dysfunction and the Foundation for Diabetes Research.

## Conflict of Interest

The authors declare that the research was conducted in the absence of any commercial or financial relationships that could be construed as a potential conflict of interest.

## Publisher’s Note

All claims expressed in this article are solely those of the authors and do not necessarily represent those of their affiliated organizations, or those of the publisher, the editors and the reviewers. Any product that may be evaluated in this article, or claim that may be made by its manufacturer, is not guaranteed or endorsed by the publisher.

## Abbreviations

DSB, double-strand-break; ICAM, intercellular adhesion molecule; IL-1β, interleukin-1 beta; IL-8, interleukin-8; NAD^+^/NADH, nicotinamide adenine dinucleotide; ROS, reactive-oxygen-species; SASP, senescence-associated-secretory phenotype; TGF-β, Transforming growth factor beta**;** TNF-α, tumor necrosis factor alpha.
